# Clinical analysis of laparoscopic pyeloplsty for ureteropelvic junction obstruction in infants under 3 months old

**DOI:** 10.3389/fped.2025.1714793

**Published:** 2026-01-05

**Authors:** Zedong Bian, Geng Xiong, Tong Liu, Yong Zhi, Ming Liu

**Affiliations:** Department of Pediatric Surgery, Affiliated Hospital of Southwest Medical University, Luzhou, Sichuan, China

**Keywords:** laparoscopy, pyeloplasty, ureteropelvic junction obstruction, infant, surgery

## Abstract

**Background:**

To evaluate the safety and efficacy of laparoscopic pyeloplasty (LP) in treating ureteropelvic junction obstruction (UPJO) among infants under 3 months of age.

**Methods:**

A retrospective analysis was performed on 45 infants with hydronephrosis caused by UPJO treated with LP between January 2019 and December 2024. Patients were divided into two groups by age: 25 infants aged 0–3 months (Group A) and 20 infants aged 4–12 months (Group B). Operative time, hospital stay, complications, anterior–posterior renal pelvic diameter (APD), and minimum renal cortex thickness (Min.RCT) were compared.

**Results:**

All procedures were completed laparoscopically without conversion to open surgery. No significant differences were observed between groups in operative time, postoperative hospital stay, complication rates, postoperative APD, or postoperative Min.RCT. Both groups showed significant improvement in APD reduction and Min.RCT increase compared with baseline. During follow-up (6–63 months, median 38.65), no recurrence or reoperation was required.

**Conclusions:**

LP is a safe and effective treatment for UPJO in infants ≤3 months with surgical indications.

## 1 Introduction

Ureteropelvic junction obstruction (UPJO) is a common cause of hydronephrosis in children (1), and Anderson-Hynes dismembered pyeloplasty is considered the gold-standard treatment (2–4). With the rapid advancement of minimally invasive techniques, laparoscopic pyeloplasty (LP) has become a widely adopted approach. Studies have affirmed its safety and efficacy for treating UPJO in children older than 4–5 months (5, 6). Despite its success, the use of LP in infants aged three months or younger, particularly neonates, remains a subject of controversy. This debate centers on the procedure's suitability and the optimal timing for surgery in this very young patient population. To address this controversy, we conducted a comparative study of 25 UPJO patients aged three months or younger and 20 patients aged four to twelve months who underwent LP at our department between January 2019 and December 2024. This study aims to evaluate the safety and efficacy of LP specifically for the treatment of UPJO in children aged three months or younger.

## 2 Materials and methods

### 2.1 Patients

A retrospective analysis was performed on the clinical data of 45 infants with hydronephrosis caused by UPJO who were admitted to the Department of Pediatric Surgery at the Affiliated Hospital of Southwest Medical University between January 2019 and December 2024. The infants were divided into two groups based on age: Group A (0–3 months old, *n* = 25) and Group B (4–12 months old, *n* = 20). In Group A, there were 19 males and 6 females, with a median age of 1.83 months. The UPJO was on the left side in 20 cases and on the right side in 5 cases. Group B consisted of 17 males and 3 females, with a median age of 6.25 months. In this group, the UPJO was on the left side in 18 cases and on the right side in 2 cases. Prenatal fetal ultrasound detected UPJO in 32 of the cases (20 in Group A, 12 in Group B). Other initial symptoms included abdominal masses (6 cases total; 2 in Group A, 4 in Group B) and urinary tract infections (7 cases total; 3 in Group A, 4 in Group B). Preoperative diagnosis of UPJO was confirmed using urinary ultrasound, computed tomography urography (CTU), and/or retrograde urography. For patients with an iodine allergy, MRI was used as an alternative to CTU and urography for further assessment of hydronephrosis severity. All patients underwent diuretic renal radionuclide scanning preoperatively, which confirmed varying degrees of urinary system obstruction. The severity of hydronephrosis was classified according to the Society of Fetal Urology (SFU) system. In Group A, one case was SFU Grade III and 24 cases were Grade IV. In Group B, two cases were SFU Grade III and 18 cases were Grade IV. There were no statistically significant differences between the two groups regarding preoperative anterior-posterior renal pelvic diameter (APD) and minimum value of renal cortex thickness (Min.RCT) (Table 1).

**Table 1:** Comparison of general data between group A and group B (mean ± SD).

Group	Gender		UPJO location		SFU classification		Preoperative APD (cm)	Preoperative Min.RCT (mm)
	Male	Female	Left	Right	III	IV		
0–3 months (*n* = 25)	19	6	20	5	1	24	4.23 ± 0.95	2.56 ± 0.91
4–12 months (*n* = 20)	17	3	18	2	2	18	4.75 ± 1.46	3.06 ± 0.91
*t* (*χ*^2^) value	*χ*^2^ = 0.141		*χ*^2^ = 0.256		*χ*^2^ = 0.040		*t* *=* −1.447	*t* = −1.819
*P* value	0.708		0.613		0.841		0.155	0.076

Patients were included if they had a confirmed preoperative diagnosis of UPJO, SFU grade III or IV hydronephrosis, no other systemic diseases, and underwent successful LP performed by the same surgeon. Patients were excluded if they had a preoperative diagnosis of ureterovesical junction obstruction (UVJO) or vesicoureteral reflux (VUR), were in the acute phase of urinary tract infection (UTI), underwent open pyeloplasty or reoperation, or had LP performed by a surgeon other than the designated operator.

### 2.2 Surgical methods

**Figure 1:**
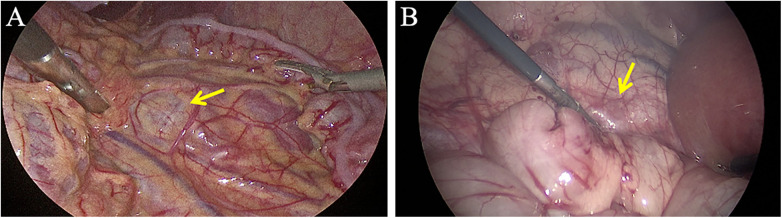
Surgical approaches for laparoscopic pyeloplasty. **(A)** For left-sided hydronephrosis, the transmesocolic approach was primarily employed. **(B)** For right-sided hydronephrosis, the lateral transcolonic approach was uniformly used. The yellow arrow indicates the dilated renal pelvis.

LP was performed with the infant in a 45° lateral decubitus position on the healthy side with an elevated waist. A pneumoperitoneum was established with a pressure of 8–10 mmHg. A 5 mm trocar was inserted at the upper edge of the umbilicus for the laparoscope. Two 3 mm trocars were placed at the lower edge of the umbilicus and the midpoint of the line connecting the umbilicus and the xiphoid process, serving as the main and auxiliary ports, respectively. For left-sided hydronephrosis, the transmesocolic approach was primarily used (Figure 1A), while the lateral transcolonic approach was uniformly used for right-sided cases (Figure 1B).

**Figure 2:**
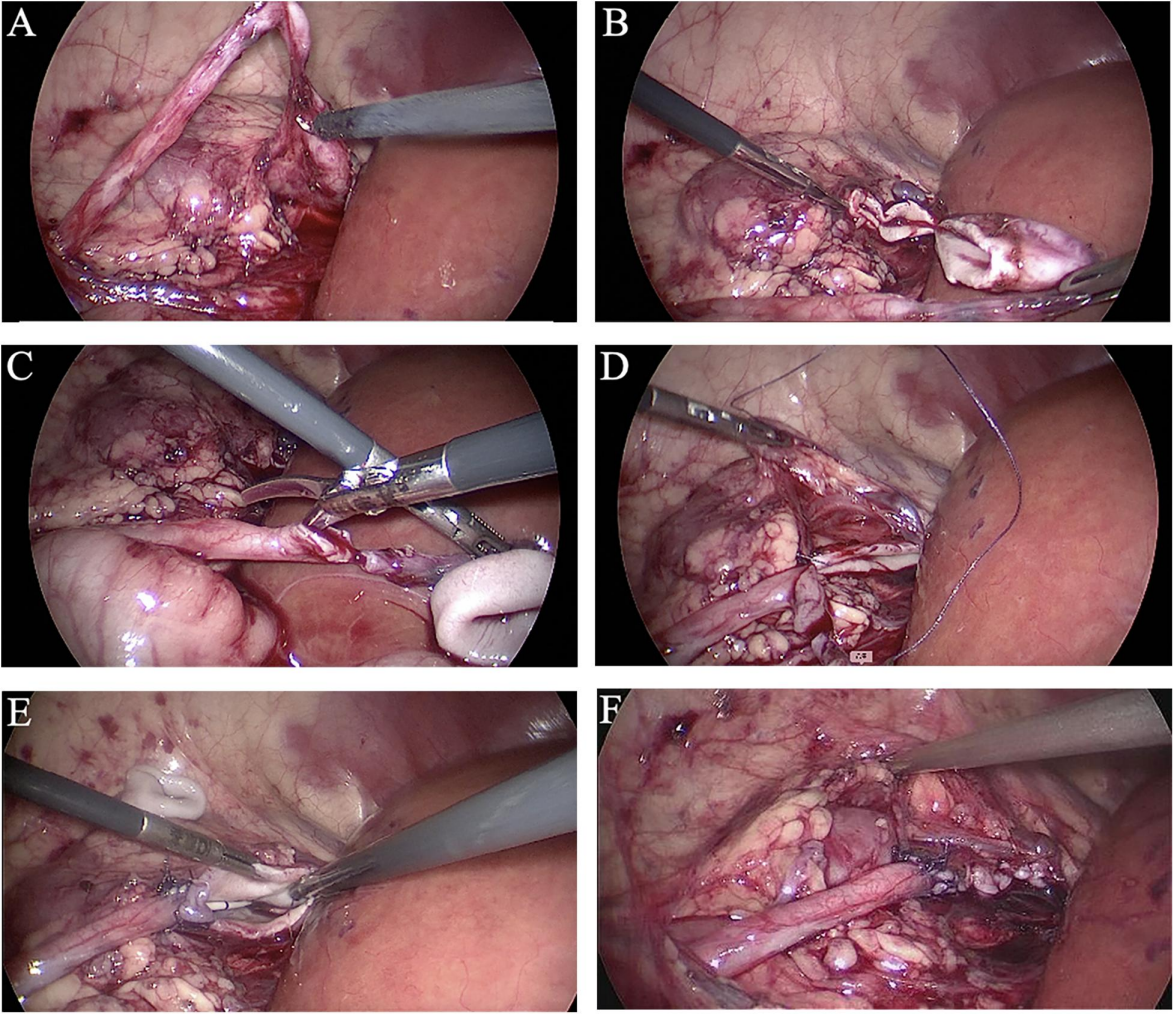
Step-by-step illustrations of laparoscopic dismembered pyeloplasty. **(A)** The ureteropelvic junction (UPJ) was exposed, and the renal pelvis and middle-upper ureter were fully dissected. **(B)** The dilated renal pelvis wall was resected approximately 1.5 cm from the renal parenchyma. **(C)** A longitudinal incision, approximately 2.0 cm long, was made on the lateral edge of the distal ureter. **(D)** The lowest points of the renal pelvis flap and the ureter were anastomosed, ensuring no tension or torsion. **(E)** An appropriately sized double-J stent was inserted through the anastomosis. **(F)** The remaining renal pelvic tissue was continuously sutured using a 4/0 absorbable barbed suture.

The retroperitoneum was incised to expose the ureteropelvic junction (UPJ) and fully dissect the renal pelvis and the middle-upper ureter (Figure 2A). The dilated renal pelvis wall was resected approximately 1.5 cm from the renal parenchyma (Figure 2B). Subsequently, a longitudinal incision, approximately 2.0 cm long, was created on the lateral edge of the distal ureter (Figure 2C). The lowest points of the renal pelvis and the ureter were anastomosed, carefully ensuring no tension or torsion (Figure 2D). A continuous side-to-side anastomosis was then performed on the anterior and posterior walls of the reshaped renal pelvis and the ureter. An appropriately sized double-J stent was inserted through the anastomosis, with its ends in the renal pelvis and bladder (Figure 2E). The remaining renal pelvic tissue was continuously sutured with a 4/0 absorbable barbed suture (Figure 2F). A perirenal drainage tube was placed below the anastomosis, and the incision was sutured in layers to conclude the operation.

### 2.3 Postoperative observation indicators

The following parameters were recorded: operative time (measured from trocar placement to completion of wound closure), requirement for conversion to open surgery, duration of postoperative hospital stay, and incidence of postoperative complications (including hematuria, urinary tract infection, urinary extravasation, and displacement or dislocation of the double-J stent). Postoperative outcomes were measured by assessing the anterior-posterior APD and Min.RCT, as well as their absolute changes (reduction in APD and increase in Min.RCT) compared to preoperative values, to evaluate surgical efficacy; no nuclear scans were performed postoperatively.

### 2.4 Statistical analysis

Statistical analyses were performed using SPSS version 22.0 (IBM, Armonk, NY, USA). Continuous variables are presented as mean ± standard deviation (SD), and categorical variables as frequencies and percentages. Intergroup comparisons of continuous variables were conducted using the independent-samples *t*-test, whereas intragroup comparisons were assessed using the paired-samples *t*-test. The Chi-square test was applied to compare categorical variables between groups. A *P* value < 0.05 was considered statistically significant.

## 3 Results

All 45 patients successfully underwent laparoscopic surgery without conversion to an open procedure. The double-J stent was removed postoperatively at 8 weeks under general anesthesia. No severe complications were observed in either group. The complications that did occur were hematuria and urinary tract infection, with no cases of urinary extravasation or double-J stent displacement. The overall complication rates were 16% for Group A and 10% for Group B, and this difference was not statistically significant (Table 2). As shown in Table 2, there were no statistically significant differences between Group A and Group B regarding operation time, which averaged 139.32 ± 16.51 min vs. 132.60 ± 10.70 min, respectively. Similarly, the postoperative hospital stay was comparable between the two groups, at 6.44 ± 1.04 days for Group A and 6.60 ± 0.99 days for Group B, with no statistically significant difference.

**Table 2:** Comparison of intraoperative and postoperative conditions between group A and group B (mean ± SD).

Group	Operation time (min)	Postoperative hospital stay (d)	Postoperative complications		
			Hematuria	Urinary tract infection	Total incidence rate (%)
0–3 months (*n* = 25)	139.32 ± 16.51	6.44 ± 1.04	1	3	16.00
4–12 months (*n* = 20)	132.60 ± 10.70	6.60 ± 0.99	1	1	10.00
*t* (*χ*^2^) value	*t* = 1.574	*t* = −0.522		*χ*^2^ = 0.086	*χ*^2^ = 0.022
*P* value	0.123	0.605	1.000^a^	0.770	0.883

a Fisher's exact test.

Postoperatively, both groups showed significant improvements in key metrics. The postoperative APD was significantly lower than the preoperative APD in both Group A (1.50 ± 0.47 cm vs. 4.23 ± 0.95 cm) and Group B (1.79 ± 0.75 cm vs. 4.75 ± 1.46 cm), indicating that urinary flow obstruction was effectively relieved (Table 3). Additionally, the postoperative Min.RCT was significantly higher in both Group A (4.60 ± 0.98 mm vs. 2.56 ± 0.91 mm) and Group B (5.17 ± 1.25 mm vs. 3.06 ± 0.91 mm) compared to preoperative values, suggesting improved kidney morphology (Table 3).

**Table 3:** Comparison of preoperative vs. postoperative APD and Min.RCT within. Group A and Group B (mean ± SD)

Time point	0–3 months (*n* = 25)		4–12 months (*n* = 20)	
	APD (cm)	Min.RCT (mm)	APD (cm)	Min.RCT (mm)
Within 1 week before surgery	4.23 ± 0.95	2.56 ± 0.91	4.75 ± 1.46	3.06 ± 0.91
6 months after surgery	1.50 ± 0.47	4.60 ± 0.98	1.79 ± 0.75	5.17 ± 1.25
*t* value	15.733	−26.263	13.319	−13.303
*P* value	0.000	0.000	0.000	0.000

There were no statistically significant differences between the groups in the reduction value of APD and the increase value of Min.RCT (Table 4). During the follow-up period, which ranged from 6 to 63 months (median of 38.65 months), no patients experienced a recurrence of urinary tract obstruction or required a reoperation.

**Table 4:** Comparison of APD and Min.RCT between group A and group B (mean ± SD).

Group	Postoperative APD (cm)	Reduction value of APD (cm)	Postoperative Min.RCT (mm)	Increase value of Min.RCT (mm)
0–3 months (*n* = 25)	1.50 ± 0.47	2.72 ± 0.86	4.60 ± 0.98	2.04 ± 0.39
4–12 months (*n* = 20)	1.79 ± 0.75	2.96 ± 0.99	5.17 ± 1.25	2.11 ± 0.71
*t* (*χ*^2^) value	−1.561	−0.851	−1.724	−0.451
*P* value	0.126	0.400	0.092	0.654

## 4 Discussion

LP is the standard surgical method for treating hydronephrosis caused by UPJO. Since its first application in children (7), LP has been widely adopted due to its minimally invasive nature, faster recovery, and comparable success rates with open surgery. However, reports of LP performed in infants remain relatively rare, and its applicability in children younger than 6 months is still debated (8, 9). With improvements in minimally invasive equipment, surgical techniques, and operator experience, accumulating evidence demonstrates that LP is safe and feasible in children weighing ≤10 kg or younger than 6 months (10–12), with high success and low complication rates similar to those observed in older children. In infants ≤3 months, however, the limited abdominal cavity and underdeveloped renal structures increase technical difficulty, and parental anxiety often complicates clinical decision-making. Thus, the safety and timing of LP in this age group remain controversial.

The primary challenge in managing congenital UPJO-induced hydronephrosis is distinguishing infants who require early surgical intervention from those suitable for conservative observation, with the goal of maximizing renal function preservation while avoiding unnecessary procedures. Previous studies have identified APD, differential renal function (DRF) <40%, SFU grades III–IV, and the presence of clinical symptoms as independent predictors for surgical intervention (13–15). Among these, APD remains a key reference indicator. Although no universally accepted cutoff exists (15–17), dynamic changes in APD provide critical evidence. In our practice, neonates with prenatal hydronephrosis are followed with urinary ultrasound every two weeks from birth to 3 months of age. Progressive APD enlargement accompanied by SFU grade III–IV changes indicates irreversible obstruction and warrants early surgical correction to minimize renal damage.

Diuretic renal radionuclide scanning is widely considered the best imaging modality for assessing renal function, and DRF <40% is frequently used as an indication for surgery (18). However, in infants ≤3 months, even in cases of partial or complete obstruction, DRF sometimes remains within the normal range. Specifically, in Group A (infants ≤3 months, *n* = 25), four cases had a DRF >40%. This may be due to high renal filtration capacity, contralateral compensation, and increased renal pelvic compliance. Therefore, DRF <40% should not be used as the sole indicator for surgery in this age group. Combining DRF and APD offers a more reliable guide for treatment decisions (15). Importantly, studies have shown that in infants with severe hydronephrosis, DRF can in certain situations improve significantly after early surgery, while delays of more than 1 month can compromise postoperative functional recovery (19). We therefore recommend early surgical intervention in infants with clear obstruction, even when DRF is ≥40%. In instances of severe hydronephrosis accompanied by a thin renal cortex, the patient's DRF must be used to determine the feasibility of kidney preservation. Our recommendation is to preserve the affected kidney whenever possible, provided that it retains some filtration function.

Patients with UPJO may also present with a complicating distal ureteral obstruction, such as vesicoureteral junction obstruction (UVJO). During pyeloplasty, this complication can prevent the insertion of the double-J stent into the bladder through the distal ureter. To mitigate this risk, we routinely confirm the patency of the distal ureter via preoperative examinations. Because ultrasound and nuclear scans cannot provide accurate morphological images of the entire ureter, we routinely perform CTU preoperatively to obtain three-dimensional images of the entire urinary tract (including the kidney, renal pelvis, ureter, and bladder) and determine the presence of distal ureteral obstruction. If CTU is inconclusive, a second-line approach involves performing cystoscopic ureteral intubation under general anesthesia, followed by retrograde urography to obtain the complete morphology of the ureter.

In our study, all infants ≤3 months underwent LP without conversion to open surgery. Operative time, postoperative hospital stay, and complication rates did not differ significantly between infants ≤3 months and those aged 4–12 months. The complication rate in Group A (16%) was similar to Group B (10%), with no severe events. Both groups exhibited significant postoperative reductions in APD and increases in Min.RCT, indicating effective relief of obstruction and structural renal improvement. No recurrences or reoperations were observed during follow-up. These findings demonstrate that LP in infants ≤3 months is both safe and effective, with outcomes comparable to older infants.

**Figure 3:**
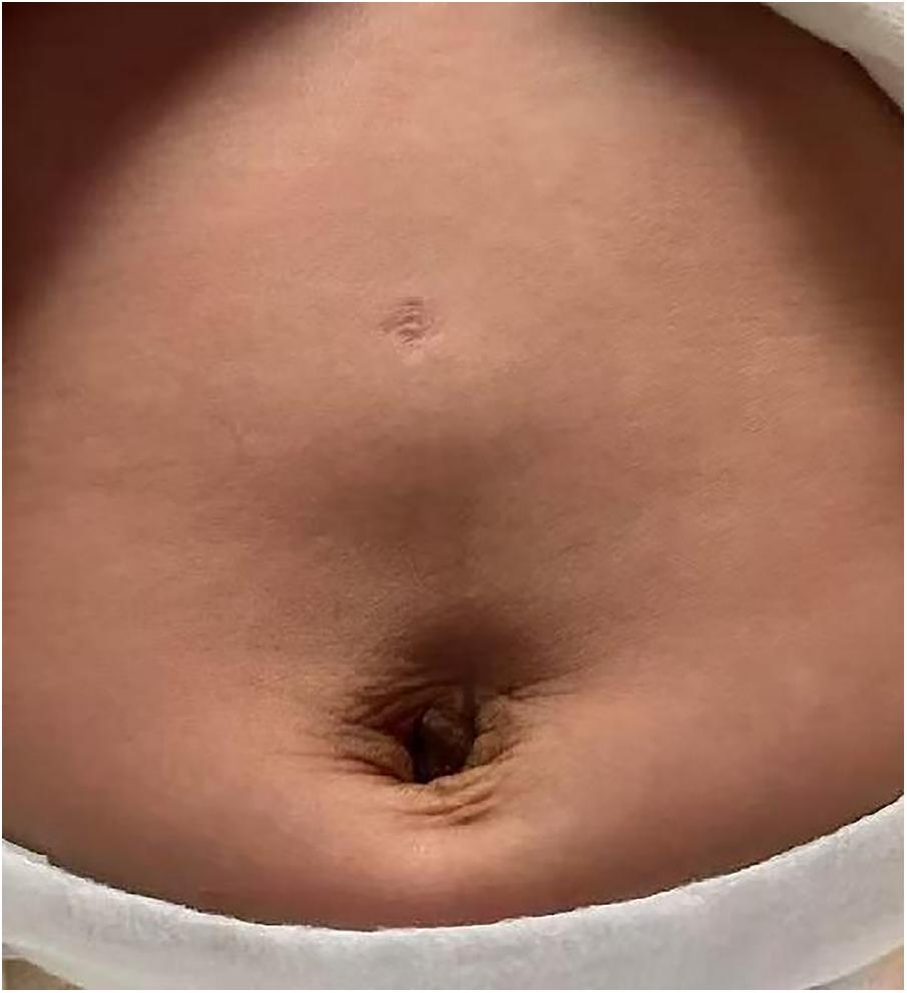
Superior postoperative cosmetic outcome. The use of a transumbilical trocar configuration allows for the concealment of scars within the natural umbilical fold, resulting in superior postoperative cosmetic outcomes.

Several technical factors are critical to achieving success in this age group. Both transperitoneal and retroperitoneal approaches have been reported with high success rates (8, 20). However, the retroperitoneal space in infants is extremely limited, increasing operative difficulty. By contrast, the transperitoneal approach provides greater working space and improved visualization, facilitating precise dissection, suturing, and knot-tying. For this reason, all infants in our series underwent LP via the transperitoneal approach. Additionally, we recommend a transumbilical single-site two-port plus one auxiliary-port trocar configuration: a 5 mm trocar at the upper umbilical edge for the laparoscope, and two 3 mm trocars at the lower umbilical edge and midway between the umbilicus and xiphoid process. This setup offers two advantages: (1) concealment of scars within the natural umbilical fold, resulting in superior cosmetic outcomes (Figure 3), and (2) reduced instrument interference compared with single-site three-port or multi-channel methods, thereby facilitating smoother procedures, shortening operative time, and reducing surgeon fatigue.

In infants ≤3 months, the narrow and tortuous ureters make double-J stent placement particularly challenging. We typically use a 3.0 French (Fr) stent with a 15 cm length, although a 4.0 French (Fr) stent of the same length may be appropriate in select cases with a relatively larger ureteral diameter. Based on our experience, several technical principles are essential: (1) advance the stent slowly along the ureteral axis to avoid excessive wall stress and prevent anastomotic dehiscence; (2) maintain appropriate bladder filling, avoiding both overdistension (which alters the ureteral angle) and overemptying (which causes mucosal folds that obstruct guidewire passage); (3) minimize repeated ureteral manipulation to reduce edema; and (4) if antegrade stent placement fails, employ retrograde placement under cystoscopy or temporary external drainage with a nephrostomy tube and ureteral catheter.

This study, however, has several limitations. Its retrospective, single-center design and relatively small sample size may limit the generalizability of our findings. Additionally, we only assessed outcomes up to a median follow-up of 38.65 months, so long-term outcomes remain to be evaluated. Larger, multicenter prospective studies are needed to confirm our results and further establish the optimal management of this patient population.

## 5 Conclusions

For infants ≤3 months with hydronephrosis caused by UPJO who meet surgical criteria, LP is a safe and effective intervention. Our findings suggest that outcomes in this age group are comparable to those in older infants. Larger multicenter prospective studies are needed to further validate these results and establish optimal surgical timing.

## Data Availability

The raw data supporting the conclusions of this article will be made available by the authors, without undue reservation.
